# Comparison of Brain Transcriptome of the Greater Horseshoe Bats (*Rhinolophus ferrumequinum*) in Active and Torpid Episodes

**DOI:** 10.1371/journal.pone.0107746

**Published:** 2014-09-24

**Authors:** Ming Lei, Dong Dong, Shuo Mu, Yi-Hsuan Pan, Shuyi Zhang

**Affiliations:** Institute of Molecular Ecology and Evolution, SKLEC & IECR, East China Normal University, Shanghai, China; Instituto Butantan, Brazil

## Abstract

Hibernation is an energy-saving strategy which is widely adopted by heterothermic mammals to survive in the harsh environment. The greater horseshoe bat (*Rhinolophus ferrumequinum*) can hibernate for a long period in the hibernation season. However, the global gene expression changes between hibernation and non-hibernation season in the greater horseshoe bat remain largely unknown. We herein reported a comprehensive survey of differential gene expression in the brain between winter hibernating and summer active greater horseshoe bats using next-generation sequencing technology. A total of 90,314,174 reads were generated and we identified 1,573 differentially expressed genes between active and torpid states. Interestingly, we found that differentially expressed genes are over-represented in some GO categories (such as metabolic suppression, cellular stress responses and oxidative stress), which suggests neuroprotective strategies might play an important role in hibernation control mechanisms. Our results determined to what extent the brain tissue of the greater horseshoe bats differ in gene expression between summer active and winter hibernating states and provided comprehensive insights into the adaptive mechanisms of bat hibernation.

## Introduction

Hibernation is a survival strategy for some mammals (e.g., bats, bears, ground squirrels) against the food deprivation in winter [1], [2]. The body temperature, metabolic rate, heart beats, and oxygen consumption of some small hibernators are remarkably reduced during hibernation [3], [4], however no neural damage is observed in the brain of hibernators after arousal from torpor [2], [5]. During the hibernation season, brain controls the initiation and end of the torpor phase, and is protected from damages induced by hypoxia-ischemia [6]. It is thought that brain regions play a pivotal role in the regulation of torpor-arousal cycle [7]–[9]. Up to date, the molecular basis for the regulation of torpor-arousal cycle has been extensively investigated in hibernators, such as the arctic ground squirrels and bears [10]–[12]. Evidences suggested that the global suppression of metabolism, protein synthesis, energy consumption, and neural excitotoxicity, as well as an increased antioxidant defense in the brain are strategies used by hibernators for neuroprotection [13]–[15]. The underlying molecular mechanisms by which hibernators prevent brain damages during hibernation may help us to develop the therapeutic strategies of human neurodegenerative disorders [16].

It has long been believed that selective gene expression plays a central role during the hibernation [17]. For example, the up-regulation of α_2-macroglobulin in the liver tissue increases the blood clotting time in squirrels of torpid state, whereas the down-regulation of prostaglandin D2 synthase in the brain tissue of hibernating squirrels is related to arousal state changes [17], [18]. Thus, gene expression variations are critical for the adaptation to change environments. Over the past decades, it has been documented that genes involved in lipolysis, non-shivering thermogenesis, and neural plasticity tend to be differentially expressed between the summer active and winter hibernating states in some animals [10], [19], [20]. Most previous researches on mammalian hibernation have performed in squirrels [10], [21], however, little is known about the molecular mechanisms in heterothermic bats.

Bats, belonging to the order Chiroptera, are one of the largest monophyletic clades in mammalian species [22]–[24]. They are the only mammals with true flight ability [25]. Most bat families contain heterothermic species, and the hibernation behavior of bats is confirmed in at least seven families [26]. Some works have been performed to screen hibernation-related genes in the brain of the greater horseshoe bat (*R. ferrumequinum*) using SSH library and dot blot [27]. The greater horseshoe bats are known to have prolific populations and use large body fat preserved as fuel to survive an annual hibernation period up to 6- to 8-months. Since bats fly immediately upon arousal and the brain plasticity has been suggested to play important roles in hibernation [18], [20], [25], it is interesting to explore the molecular mechanisms underlying brain function and protection during bat torpor. Thanks to the emergence of next-generation sequencing technology, which opens the door for us to obtain cDNA fragments from transcriptome with reasonably complete coverage in a reduced time scale and at a lower cost [28]. In this study, we performed next-generation sequencing to compare the brain transcriptomes of active and torpid greater horseshoe bats and to identify differentially expressed genes that contribute to the hibernation phenotype.

## Materials and Methods

### Brain tissue sample preparation and deep sequencing

All animals used in this work were carried out in strict accordance with the approval of Regulations for the Administration of Laboratory Animals (Decree No. 2 of the State Science and Technology Commission of the People’s Republic of China on November 14, 1988) approved by the Animal Ethics Committee of East China Normal University (ID no: 20090219 and 20101002). Six male summer active and six male winter torpid greater horseshoe bats were wild-caught in the Fish cave (30°20′N, 117°50′E) in Anhui Province, China on June 24 (summer active state), 2009, and December 11 (winter torpid state), 2009, respectively. No specific permissions were required for the greater horseshoe bats capture in the Fish cave. The surface temperatures of the active and torpid bats were approximately 25°C and 5°C, respectively. The greater horseshoe bats were euthanized by respiratory hyperanesthesia followed thoracotomy, and all efforts were made to minimize potential pain and suffering. The whole brain tissues (without cerebellum) were stored in RNAlater solution (Takara Biotechnology Co. Ltd., Dalian, China) to preserve the RNA state for use immediately after killing.

Total RNA was extracted using Trizol (Life Technologies Corp.) according to the manufacturer’s protocols and treated with RNeasy Mini kit (Qiagen). RNA concentration was evaluated using a NanoDrop spectrophotometer and RNA quality was assessed by Agilent Bioanalyzer. Total RNA was submitted to majorbio company, Shanghai, China for deep sequencing using an Illumina Genome Analyzer II platform. Two lanes per group were sequenced as 75-bp reads. Raw sequence data generated by Illumina pipeline were deposited into Short Read Archive (SRA) database of NCBI with the accession no. SRR1048140 (summer active state) and SRR1048142 (winter torpid state), and all assembled contigs were deposited into NCBI TSA database with the accession no. GAVY01000000.

### Data preprocess and *de novo* transcriptome assembly

To reduce sequencing errors, DynamicTrim Perl script implemented in SolexQA package [29] was performed to control the quality of raw sequencing data with the default parameters setting. Next, we *de novo* assembled the transcriptome sequences using Trinity package [30]. The assembled contigs with length less than 200 bp were discarded due to their low annotation rate. The program was run on 64-bit Linux system (Red Hat 6.0) with 256 GB internal memory. After transcriptome assembly, CD-Hit program [31] was then used to reduce sequence redundancy of transcriptome with default parameter setting.

### Function annotation and RNA-Seq analysis

As for the functional annotation, assembled contigs were searched by BLASTX against the NCBI non-redundant nucleotide database with an E-value threshold of 1E-5. The results of the best blast hits were extracted, and coding sequences were subsequently determined. Functional annotation was implemented using the online software Blast2GO [32]. Gene ontology (GO) terms and annotations were downloaded on May 22, 2012. These identified genes were classified into different levels of GO categories.

We then estimated read counts of every gene using RSEM [33] nested in the Trinity package [30]. To compare the gene expression difference between the active and torpid states, we normalized the read counts by calculating FPKM (fragments per kilobase of exon per million fragments mapped) values. In this work, EdgeR method was used to detect differentially expressed genes [34]. After applying Benjamini-Hochberg correction for multiple test, the false discovery rate (*FDR*) <0.05 and fold change greater than 2 were selected as the criteria for significant differences. In order to explore the biological implications, all differentially expressed genes were submitted to DAVID online toolkit [35].

## Results

### *De novo* assembly and functional annotation

**Figure 1:**
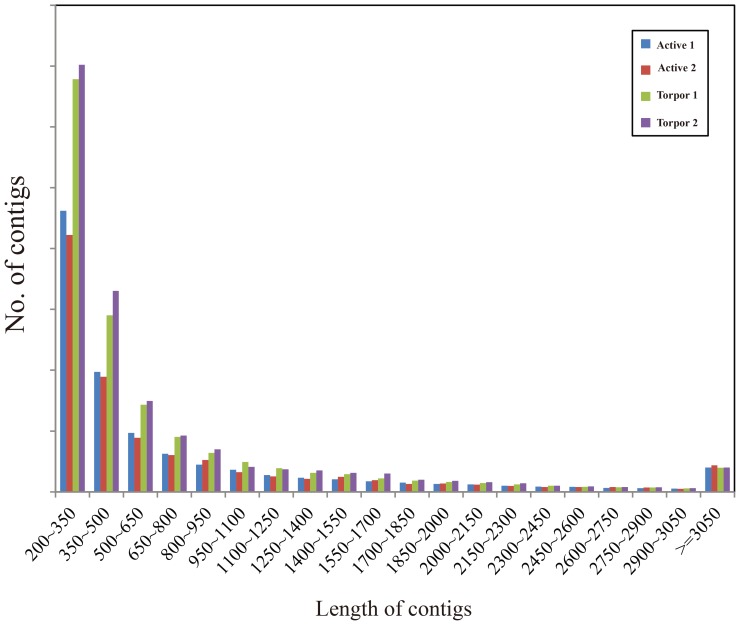
Length distribution of greater horseshoe bat transcriptome.

**Figure 2:**
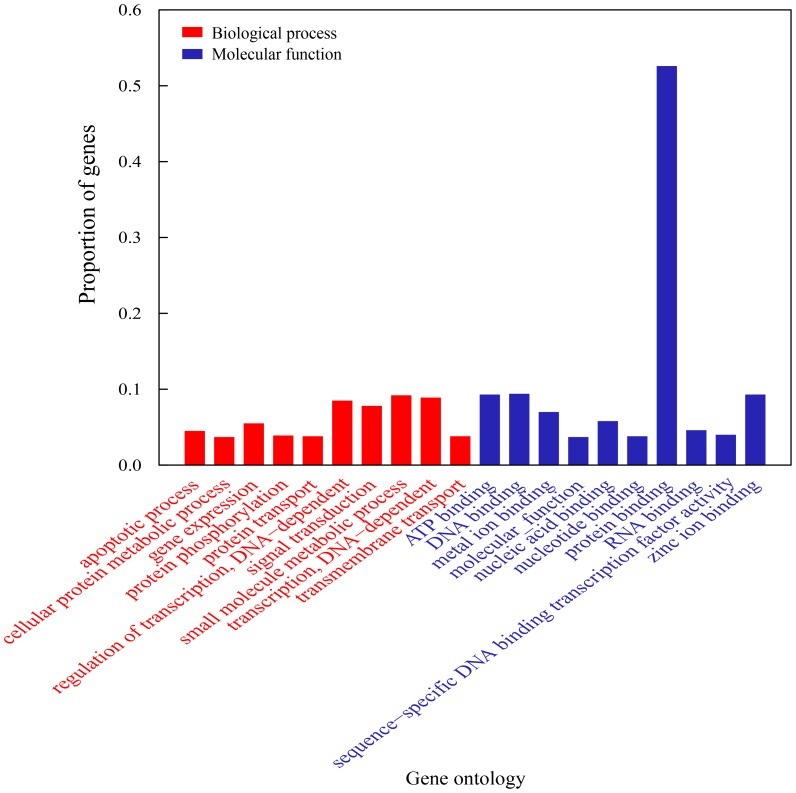
Top 10 represented GO categories of biological process (red bars) and molecular function (blue bars).

To obtain an overview of brain transcriptome of summer active and winter torpid greater horseshoe bat, cDNA samples were prepared from mixtures of RNA from the whole brain (without cerebellum) and then were sequenced using Illumina Genome Analyzer II. Each sample group was sequenced in two lanes, and a total of 51,755,965 and 38,558,209 paired-end reads with 75-bp length for active and torpid samples were generated (**Table 1**). After cleaning of raw sequences, the trimmed reads were assembled using Trinity package [30]. As shown in **Figure 1**, contigs with their lengths between 200 and 350 bp were overrepresented, making up ∼38% of the total number of contigs. We even identified 7,445 contigs longer than 3,050 bp. Next, we used CD-Hit program [31] to generate non-redundant contigs. A total of 11,279 contigs with 95% sequence identity were removed, and only the longest one was retained if more than one contigs correspond to a gene. Finally, a total of 11,214 genes were obtained representing about 10% of all distinct contigs. Of these annotated genes, 9,910 (93%) were assigned to at least one GO categories and small molecule metabolic process (GO:0044281) and protein binding (GO:0005515) were most represented (**Figure 2** **, Table S1**).

**Table 1:** *De novo* assembly statistics.

	Active 1	Active 2	Torpor 1	Torpor 2
Total number of reads	29,066,538	22,689,427	16,878,030	21,650,179
Total number of filtered reads	28,752,432	21,055,685	16,682,052	21,172,836
Total number of used reads	22,536,772	17,461,080	12,813,908	17,347,136
Number of contigs	61,863	60,601	78,893	69,461
N50	1,776	1,472	1,107	1,402
Max. Length	14,968	12,852	11,781	12,077
Ave. length	950	886	703	798

Note: N50 represents the median contig size of our transcriptome assembly.

### Differential expression analysis

FPKM (fragments per kilobase of exon per million fragments mapped) values were used as proxies of gene expression level. To investigate the expression patterns of the active and torpid samples of the greater horseshoe bats, we evaluated the number of reads assembled from each library for each transcript and compared gene expression difference between them. In this work, edgeR method [34] was used to measure gene expression differences between summer active and winter torpid bats. A total of 1573 genes were identified to be significantly differentially expressed between these summer active and winter torpid samples with the criteria of *F.D.R* <0.05 and fold change >2 (**Table S2**), within which 1038 genes are up-regulated in the torpid state and 535 genes are down-regulated in the torpid state. Among these differentially expressed genes, the gene with the most significant (*P-value* = 3.89E-202) expression difference between the active and torpid samples is heat shock 70 kDa protein 8 (*HSPA8*), with a ∼4.46 fold higher expression in the torpor sample. *HSPA8* belongs to the heat-shock cognate subgroup, which functions as an ATPase working with auxilin to remove clathrin coated vesicles [36]. Conversely, the gene with the most significant (*P-value* = 4.77E-154) expression difference and higher expression in the active sample (6.01 fold) is *CKB*, which encodes a cytoplasmic enzyme involved in energy homeostasis [37]. **Table 2** lists the top 10 differentially expressed genes that were significantly altered between active and torpid samples.

**Table 2:** Top 10 up- and down-regulated genes in the torpid brains.

	Gene Symbol	Description	Average FPKM (Torpor)	Average FPKM (Active)	*F.D.R*
**Up-regulated genes in torpid brain**	HSPA8	Heat shock 70 kDa protein 8	1344.595	300.3264	3.89E-202
	SNAP25	Synaptosomal-associated protein, 25 kDa	862.1088	101.904	1.98E-184
	CALM1	Calmodulin	991.6896	318.3168	9.56E-109
	CDR1	Cerebellar degeneration-related antigen 1	337.44	11.1648	7.29E-100
	HSP90AA1	Heat shock protein HSP 90-alpha	444.6336	63.984	1.58E-87
	CPE	Carboxypeptidase E	476.5056	91.2192	1.95E-80
	SYT1	Synaptotagmin-1	292.0512	29.4048	5.18E-67
	SPARCL1	SPARC-like protein 1	253.056	20.9856	2.32E-62
	RPS24	40S ribosomal protein S24	432.9888	117.6096	1.51E-56
	CAMK2N1	Calcium/calmodulin-dependent protein kinase II inhibitor 1	463.248	144.192	1.41E-53
**Down-regulated genes in torpid brain**	CKB	Creatine kinase, brain	207.0912	1240.042	4.77E-154
	ALDOA	Aldolase A, fructose-bisphosphate	427.1424	1679.405	1.07E-142
	ALDOC	Aldolase C, fructose-bisphosphate	491.472	1656.365	1.54E-116
	TUBA1B	Tubulin, alpha 1b	510.6336	1607.155	1.16E-102
	MBP	Myelin basic protein	71.7408	613.7568	3.01E-95
	ATP1A3	ATPase, Na+/K+ transporting, alpha 3 polypeptide	184.368	874.7904	1.13E-90
	NDRG2	N-myc downstream-regulated gene 2 protein	177.744	797.7216	9.42E-79
	APOE	Apolipoprotein E	377.4912	1187.52	2.78E-76
	RPS28	40S ribosomal protein S28	181.6128	770.9184	4.17E-72
	TUBA4A	Tubulin alpha-4A chain	179.5488	760.1952	9.34E-71

### Gene ontology enrichment analysis

In order to gain insights into the biological implications of these differentially expressed genes, we performed the GO enrichment analysis. A total of 261 GO categories belonging to molecular function and biology process sub-categories were identified. The significant overrepresented GO terms were listed in **Table S3**, and the enriched GO terms of significantly up-regulated or down-regulated genes in the torpid brain were all listed in **Table S4**. In these GO categories, those responsible for energy metabolism, cellular stress responses, oxidative stress, and cytoskeleton organization were observed to be overrepresented during bat torpor. Some important functional categories are summarized below.

#### Metabolism

**Figure 3:**
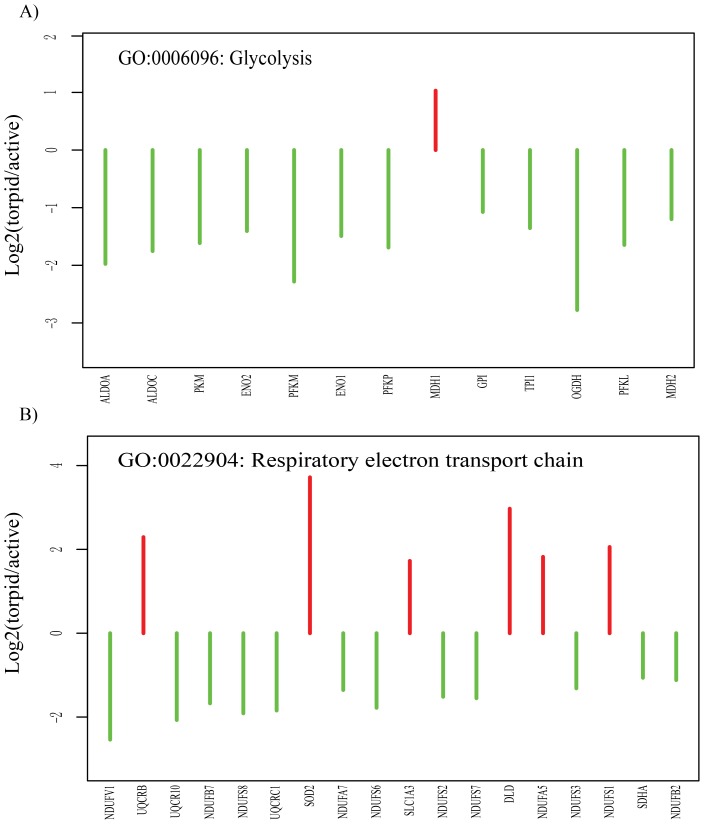
Genes that regulate energy metabolism (A respiratory electron transport chain and B glycolysis) are significantly up- or down- regulated in the torpid brain. The red and green lines represent the fold change values of genes up- and down regulated in the torpid brain, respectively. The genes are ordered according to their *P-values*.

The genes involved in ‘glycolysis’ and ‘respiratory electron transport chain’ were significantly down-regulated in torpid bats. As shown in **Figure 3**, glycolytic enzymes including phosphofructokinase 1 (*PFKP*, *PFKM*, *PFKL*), fructose-bisphosphate aldoase (*ALDOA*, *ALDOC*), enolase (*ENO1*, *ENO2*), glucose phosphate isomerase (*GPI*), pyruvate kinase (*PKM*) have a low expression level during bat torpor. Of these, Phosphofructokinase 1 and pyruvate kinase are known as key rated-limited regulated enzymes of glycolysis. NADH dehydrogenase is a complicated oxidoreductase, which catalyzes the transfer of electrons from NADH to coenzyme Q, and plays an important role in the mitochondrial respiration. NADH dehydrogenase (ubiquinone) subunits containing NADH dehydrogenase (ubiquinone) Fe-S protein (*NDUFS2*, *NDUFS3*, *NDUFS6*, *NDUFS7*, *NDUFS8*), NADH dehydrogenase (ubiquinone) 1 beta subcomplex (*NDUFB2*, *NDUFB7*), NADH dehydrogenase (ubiquinone) 1 alpha subcomplex (*NDUFA7*), and NADH dehydrogenase (ubiquinone) flavoprotein 1 (*NDUFV1*) were down-regulated in the torpid brain.

#### Cellular stress responses

**Figure 4:**
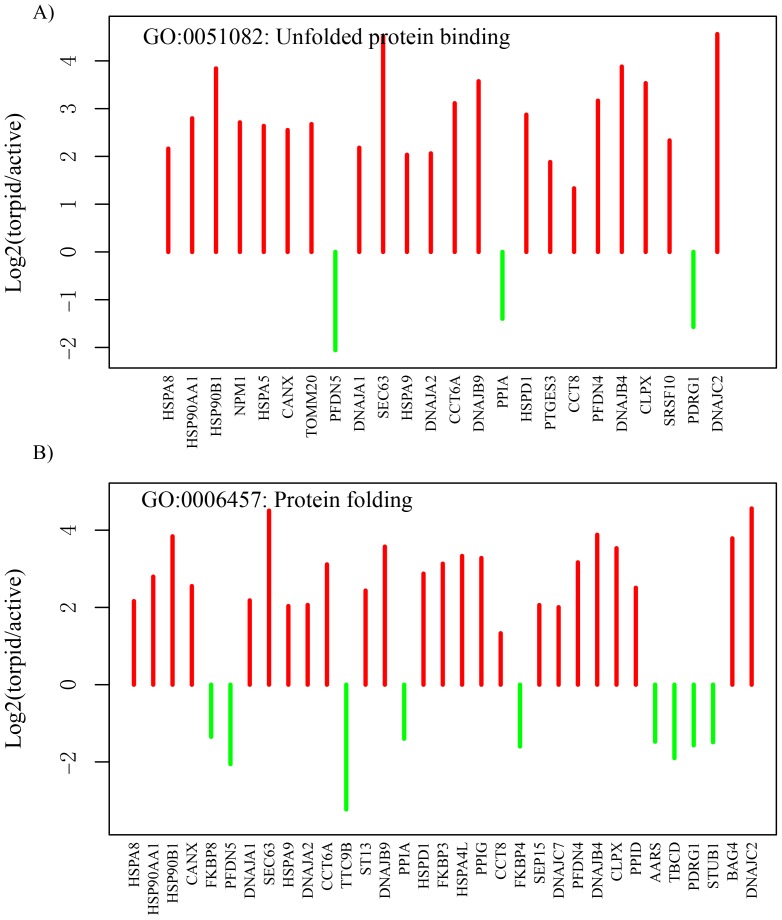
Genes involved in responses to cellular stress (A unfolded protein binding” and B protein folding) are significantly up- or down- regulated in the torpid brain. The red and green lines represent the fold change values of genes up- and down regulated in the torpid brain, respectively. The genes are ordered according to their *P-values*.

GO categories of ‘unfolded protein binding’ and ‘protein folding’ are examples representing the cytoplasmic stress response. Most of the genes involving these GO categories were over-overexpressed during torpor (**Figure 4**). We found many heat shock proteins (HSPs) were differentially expressed. HSPs, a group of proteins induced by heat shock, play diverse roles in folding, regulation and degradation of other proteins; repair of DNA and chromatin and cell cycle control in response to outside stresses [38], [39]. The differentially expressed HSP families include Hsp70 (*HSPA8*, *HSPA5*, *HSPA9*, *HSPA4L*), Hsp90 (*HSP90AA1*, *HSP90B1*) and Hsp40 (*DNAJA1*, *DNAJA2*, *DNAJB4*, *DNAJB9*, *DNAJC2*, *DNAJC7*) and Hsp60 (*HSPD1*), which have high level in expression suggesting an adaptive response of torpid bats to environmental stresses.

#### Oxidative stress

**Figure 5:**
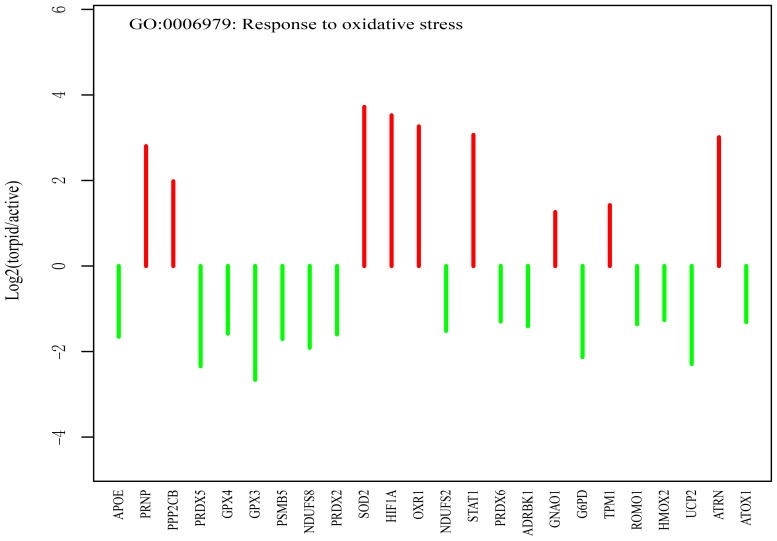
Genes related to oxidative stress (*response to oxidative stress*) are significantly up- or down-regulated in the torpid brain. The red and green lines represent the fold change values of genes up- and down-regulated in the torpid brain, respectively. The genes are ordered according to their *P-values*.

Oxidative stress is an imbalance between free radicals and cellular antioxidant defense, which can lead to cell death by damaging proteins, nucleic acids and lipids through free radicals or peroxides [40]. Genes involved in GO category of ‘response to oxidative stress’ are mainly down-regulated during the hibernation (**Figure 5**). For example, the glutathione peroxidase 3 (*GPX3*) gene, which functions in the detoxification of hydrogen peroxide, has a ∼8 fold lower expression in the torpid brain compared with the active brain.

#### Stabilization of microtubule

**Figure 6:**
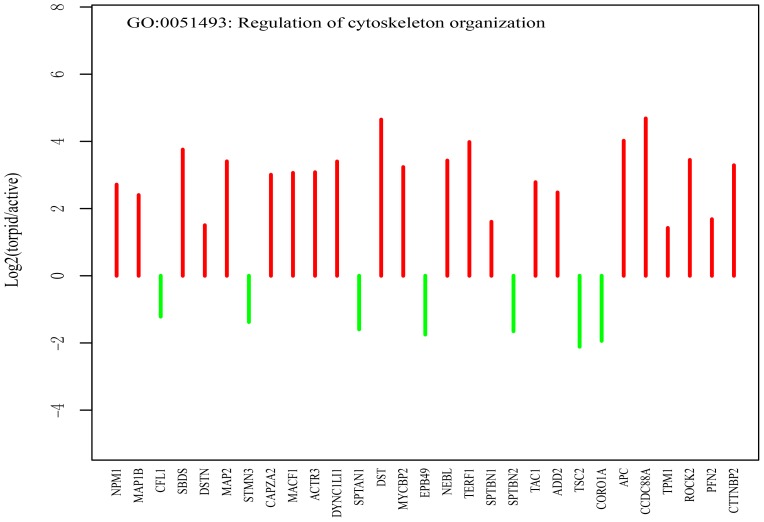
Genes related to stabilization of cytoskeleton (regulation of cytoskeleton organization). The red lines represent the fold change values of genes up-regulated in the torpid brain, and the green lines represent the fold change values of genes up-regulated in the active brain. The genes are ordered according to their *P-values*.

Next, GO term of ‘regulation of cytoskeleton organization’ was enriched among differentially expressed genes between active and torpid samples (**Figure 6**). For example, two neural microtubules-associated protein genes (*MAP1B* and *MAP2*) which cooperatively play important roles in dendritic outgrowth and microtubule organization [41] show elevated expression during torpor. Previously, Chen *et al.* also found that MAP1B up-regulated in the torpid brain [27]. In addition, three cytoskeletal genes, dystonin (*DST*), microtubule-actin cross-linking factor 1 (*MACF1*) and adenomatous polyposis coli protein (*APC*), are also overexpressed during the hibernation. These genes might be important for the characterization of neural morphology in the torpid brain.

## Discussion

Animal hibernation is an adaptive and complex process of metabolic suppression for energy conservation in winter. To understand the molecular mechanisms of mammalian hibernation, some genomic studies have been conducted using squirrels and bears as models. However, the transcriptomic changes in response to hibernation in bats remain largely unknown. In this work, we compared the brain transcriptome of greater horseshoe bats between active and torpid states.

Hibernating bats can preserve fat during pre-hibernation period as a primary energy source for surviving in the hibernation season. The concentration of blood glucose is low during bat torpor [42]. The brain prefers to utilize ketones rather than glucose as the primary energy source during hibernation. [43]. Glycolysis, a key metabolic pathway of glucose catabolism, includes 10 sequential enzyme reactions to convert glucose into pyruvate and prepares for energy production. The low expression of nine genes (i.e., *PFKP*, *PFKM*, *PFKL*, *ALDOA*, *ALDOC*, *ENO1*, *ENO2, GPI*, and *PKM*) involved in glycolysis of torpid bats supports the theory of glucose conservation during mammalian torpor. In addition to the conservation of glucose, down-regulation of glycolytic pathway plays a central role in metabolic suppression during torpor. The co-downregulation of these genes in the torpid bats further suggests a global suppression of metabolism at bat torpor, however, malate dehydrogenase 1 (MDH1), which produces oxaloacetate for gluconeogenesis, was elevated during torpor. This may be a continual pathway that prepares glucose for utilization of bats upon arousal. Most of the NADH dehydrogenase (ubiquinone) subunits were down-regulated in torpid bats suggested that electron transport is also suppressed during hibernation. Nonetheless, the protein subunits such as NDUFS1 and NDUFA5 were elevated in expression. This seems to agree with the up-regulation of some subunits of mitochondrial membrane proteins (e.g., NDUFS1, NDUFS3, NDUFV2) in brainstem of ground squirrels at earlier torpor, suggesting the common strategies adopted by small hibernators (i.e., bats and squirrels) in control of NADH dehydrogenase activity [44]. In the mitochondria, electron transport promotes ATP synthesis by generating electrochemical gradient across membranes in the aerobic environment. The results described above strongly suggest that a reduction of electron transport occurs in the brain of torpid bats to adapt low oxygen supply and to reduce the reactive oxygen species (ROS) production. Therefore, a global metabolic suppression is achieved.

Protein synthesis and proteolysis were suppressed during the torpor [3], [45]. Our findings of the elevated expression of multi-family HSP (heat shock proteins) genes suggested the preservation of proteome by protein folding during bat torpor. Stresses, such as hypoxia or ischemia, induce protein mis-folding during animal hibernation, which also occur in the pathological conditions related to neurodegenerative diseases and brain injury. HSPA8, a highly abundant member of HSP70s, was an important factor for protein refolding, which reduces the neuronal loss in the neurodegenerative disease [46]. Moreover, overexpression of two mitochondrial HSPs (HSPA9, HSPD1) increases the brain resistance to oxidative stress from focal ischemia [47], [48]. In addition, the overexpression of Hdj-2 (DNAJA1, HSP70 partner) could reduce the ischemia-like injury [49]. Elevated expression of HSPs in various tissue of hibernating animals shows that HSPs play specific role in protein homeostasis to maintain tissue specific functions [50]–[52]. In our study, the elevated expressions of multiply families of HSP gene may provide a potential mechanism for synergistically protecting the nervous system during bat torpor.

During the hibernation season, ischemic/reperfusion switch in the brain leads to oxidative stress [53], [54]. Several proteins related to antioxidant defense are shown to be elevated in the torpid brain of bats [55]. PRNP is a prion protein that promotes the activity of superoxide dismutase 1 (SOD1). It has been documented that knock-out of this gene results in increased levels of oxidative stress in the brain [56], [57]. Therefore, elevated expression level of PRNP may increase the antioxidant defense in the torpid brain. Because PRNP has diverse vital roles in nervous system and tumorigenesis [58], [59], we hypothesized that up-regulation of PRNP is involved in the maintenance of neural morphology and anti-apoptosis during the torpor. Superoxide dismutase, an important antioxidant, eliminates free radicals in the body. Up-regulation of SOD2 could protect the neural cell from free radical damage in the torpor. Yan *et al.* also found the elevated levels of SOD2 in the brown adipose tissue of hibernating arctic ground squirrels [10]. Besides the typical antioxidant, Oliver *et al.* suggested that oxidation resistance protein 1 gene (*OXR1*) is essential for the protection of neurons from oxidative stresses [60]. Increased expressions of OXR1 enhance the sensitivity of neurons under stress conditions. In this study, we also found an elevated expression of the OXR1 gene in the torpid brain of bat.

During the hibernation season, hibernators undergo global temperature-mediated neural plasticity where dendrites, cell bodies and spines significantly retract in the torpor [61], [62]. The differentially expressed cytoskeletal gene influences the stabilization of cytoskeletal network leading to the neural plasticity [20]. Although neural plasticity protects the brain, significant changes of neural structure can result in memory loss [61], [63]. However, it has been shown that bats keep their long term memory of food-finding after hibernation [64]. In this study, the expressions of two microtubule stabilizers (i.e., *MAP1* and *MAP2*) are found to be elevated in the torpid bat brain. Previous work suggested that a lower MAP2 gene expression reduces the microtubule density in dendrites and length of dendrites [65]. Arendt *et al.* also showed that a lower MAP2 density enhances the dendritic retraction due to reduction of the stable microtubules during the torpor [66]. These findings suggest that bats may reduce retraction of dendrites by stabilizing the microtubule in the dendrites through elevating MAP2 gene expression. Although the neuronal capacity is significantly reduced, hibernating mammals are capable of sensing periodical arousals and environmental stimulations [55]. Therefore, the highly expressed *SNAP25* and *SYT1* (involved in vesicle docking and synaptic transmission) identified in torpid bats suggest that the neural plasticity occurs in the brain at bat torpor.

In conclusion, we have detected hibernation-related genes in the brain of greater horseshoe bats by deep transcriptome sequencing. We have also identified differential expression of genes involved in glycolysis, respiratory electron transport chain, unfolded protein binding, protein folding, oxidative stress, and cytoskeleton organization. Results of this study will further our understanding on the molecular mechanism of mammalian hibernation.

## Supporting Information

Table S1 **Top 10 represented GO categories of biological process and molecular function.**
(XLSX)

Table S2 **The complete average FPKM values in active and torpor bat brain.**
(XLSX)

Table S3 **Over-represented GO categories in the differentially expressed genes between active and torpor bat brain.**
(XLSX)

Table S4 **Over-represented GO categories in the significantly up- and down-regulated genes.**
(XLSX)

## References

[1] Geiser F (2013) Hibernation. Curr Biol 23: R188–193. 23473557 10.1016/j.cub.2013.01.062

[2] Carey HV, Andrews MT, Martin SL (2003) Mammalian hibernation: cellular and molecular responses to depressed metabolism and low temperature. Physiol Rev 83: 1153–1181. 14506303 10.1152/physrev.00008.2003

[3] Storey KB, Storey JM (2004) Metabolic rate depression in animals: transcriptional and translational controls. Biol Rev Camb Philos Soc 79: 207–233. 15005178 10.1017/s1464793103006195

[4] Geiser F (2004) Metabolic rate and body temperature reduction during hibernation and daily torpor. Annu Rev Physiol 66: 239–274. 14977403 10.1146/annurev.physiol.66.032102.115105

[5] Frerichs KU, Kennedy C, Sokoloff L, Hallenbeck JM (1994) Local cerebral blood flow during hibernation, a model of natural tolerance to “cerebral ischemia”. J Cereb Blood Flow Metab 14: 193–205. 8113316 10.1038/jcbfm.1994.26

[6] Frerichs KU, Hallenbeck JM (1998) Hibernation in ground squirrels induces state and species-specific tolerance to hypoxia and aglycemia: an in vitro study in hippocampal slices. J Cereb Blood Flow Metab 18: 168–175. 9469159 10.1097/00004647-199802000-00007

[7] Drew KL, Buck CL, Barnes BM, Christian SL, Rasley BT, et al (2007) Central nervous system regulation of mammalian hibernation: implications for metabolic suppression and ischemia tolerance. J Neurochem 102: 1713–1726. 17555547 10.1111/j.1471-4159.2007.04675.x PMC3600610

[8] Pakhotin PI, Pakhotina ID, Belousov AB (1993) The study of brain slices from hibernating mammals in vitro and some approaches to the analysis of hibernation problems in vivo. Prog Neurobiol 40: 123–161. 8430211 10.1016/0301-0082(93)90021-j

[9] Milsom WK, Zimmer MB, Harris MB (1999) Regulation of cardiac rhythm in hibernating mammals. Comp Biochem Physiol A Mol Integr Physiol 124: 383–391. 10682236 10.1016/s1095-6433(99)00130-0

[10] Yan J, Burman A, Nichols C, Alila L, Showe LC, et al (2006) Detection of differential gene expression in brown adipose tissue of hibernating arctic ground squirrels with mouse microarrays. Physiol Genomics 25: 346–353. 16464973 10.1152/physiolgenomics.00260.2005

[11] Fedorov VB, Goropashnaya AV, Toien O, Stewart NC, Chang C, et al (2011) Modulation of gene expression in heart and liver of hibernating black bears (Ursus americanus). BMC Genomics 12: 171. 21453527 10.1186/1471-2164-12-171 PMC3078891

[12] Fedorov VB, Goropashnaya AV, Toien O, Stewart NC, Gracey AY, et al (2009) Elevated expression of protein biosynthesis genes in liver and muscle of hibernating black bears (Ursus americanus). Physiol Genomics 37: 108–118. 19240299 10.1152/physiolgenomics.90398.2008 PMC3774579

[13] Dave KR, Christian SL, Perez-Pinzon MA, Drew KL (2012) Neuroprotection: lessons from hibernators. Comp Biochem Physiol B Biochem Mol Biol 162: 1–9. 22326449 10.1016/j.cbpb.2012.01.008 PMC3334476

[14] Nathaniel TI (2008) Brain-regulated metabolic suppression during hibernation: a neuroprotective mechanism for perinatal hypoxia-ischemia. Int J Stroke 3: 98–104. 18706003 10.1111/j.1747-4949.2008.00186.x

[15] Drew KL, Rice ME, Kuhn TB, Smith MA (2001) Neuroprotective adaptations in hibernation: therapeutic implications for ischemia-reperfusion, traumatic brain injury and neurodegenerative diseases. Free Radic Biol Med 31: 563–573. 11522441 10.1016/s0891-5849(01)00628-1

[16] Zhou F, Zhu X, Castellani RJ, Stimmelmayr R, Perry G, et al (2001) Hibernation, a model of neuroprotection. Am J Pathol 158: 2145–2151. 11395392 10.1016/S0002-9440(10)64686-X PMC1891987

[17] Srere HK, Wang LC, Martin SL (1992) Central role for differential gene expression in mammalian hibernation. Proc Natl Acad Sci U S A 89: 7119–7123. 1379733 10.1073/pnas.89.15.7119 PMC49657

[18] O’Hara BF, Watson FL, Srere HK, Kumar H, Wiler SW, et al (1999) Gene expression in the brain across the hibernation cycle. J Neurosci 19: 3781–3790. 10234010 10.1523/JNEUROSCI.19-10-03781.1999 PMC6782720

[19] Williams DR, Epperson LE, Li W, Hughes MA, Taylor R, et al (2005) Seasonally hibernating phenotype assessed through transcript screening. Physiol Genomics 24: 13–22. 16249311 10.1152/physiolgenomics.00301.2004

[20] Schwartz C, Hampton M, Andrews MT (2013) Seasonal and regional differences in gene expression in the brain of a hibernating mammal. PLoS One 8: e58427. 23526982 10.1371/journal.pone.0058427 PMC3603966

[21] Hampton M, Melvin RG, Kendall AH, Kirkpatrick BR, Peterson N, et al (2011) Deep sequencing the transcriptome reveals seasonal adaptive mechanisms in a hibernating mammal. PLoS One 6: e27021. 22046435 10.1371/journal.pone.0027021 PMC3203946

[22] Teeling EC, Springer MS, Madsen O, Bates P, O’Brien SJ, et al (2005) A molecular phylogeny for bats illuminates biogeography and the fossil record. Science 307: 580–584. 15681385 10.1126/science.1105113

[23] Wilkinson GS, South JM (2002) Life history, ecology and longevity in bats. Aging Cell 1: 124–131. 12882342 10.1046/j.1474-9728.2002.00020.x

[24] Jones G, Teeling EC (2006) The evolution of echolocation in bats. Trends Ecol Evol 21: 149–156. 16701491 10.1016/j.tree.2006.01.001

[25] Ransome R (1990) The natural history of hibernating bats: Helm London, United Kingdom.

[26] Geiser F, Stawski C (2011) Hibernation and torpor in tropical and subtropical bats in relation to energetics, extinctions, and the evolution of endothermy. Integr Comp Biol 51: 337–348. 21700575 10.1093/icb/icr042

[27] Chen J, Yuan L, Sun M, Zhang L, Zhang S (2008) Screening of hibernation-related genes in the brain of Rhinolophus ferrumequinum during hibernation. Comp Biochem Physiol B Biochem Mol Biol 149: 388–393. 18055242 10.1016/j.cbpb.2007.10.011

[28] Wang Z, Gerstein M, Snyder M (2009) RNA-Seq: a revolutionary tool for transcriptomics. Nat Rev Genet 10: 57–63. 19015660 10.1038/nrg2484 PMC2949280

[29] Cox MP, Peterson DA, Biggs PJ (2010) SolexaQA: At-a-glance quality assessment of Illumina second-generation sequencing data. BMC Bioinformatics 11: 485. 20875133 10.1186/1471-2105-11-485 PMC2956736

[30] Grabherr MG, Haas BJ, Yassour M, Levin JZ, Thompson DA, et al (2011) Full-length transcriptome assembly from RNA-Seq data without a reference genome. Nat Biotechnol 29: 644–652. 21572440 10.1038/nbt.1883 PMC3571712

[31] Li W, Godzik A (2006) Cd-hit: a fast program for clustering and comparing large sets of protein or nucleotide sequences. Bioinformatics 22: 1658–1659. 16731699 10.1093/bioinformatics/btl158

[32] Conesa A, Gotz S (2008) Blast2GO: A comprehensive suite for functional analysis in plant genomics. Int J Plant Genomics 2008: 619832. 18483572 10.1155/2008/619832 PMC2375974

[33] Li B, Dewey CN (2011) RSEM: accurate transcript quantification from RNA-Seq data with or without a reference genome. BMC Bioinformatics 12: 323. 21816040 10.1186/1471-2105-12-323 PMC3163565

[34] Robinson MD, McCarthy DJ, Smyth GK (2010) edgeR: a Bioconductor package for differential expression analysis of digital gene expression data. Bioinformatics 26: 139–140. 19910308 10.1093/bioinformatics/btp616 PMC2796818

[35] Huang da W, Sherman BT, Lempicki RA (2009) Systematic and integrative analysis of large gene lists using DAVID bioinformatics resources. Nat Protoc 4: 44–57. 19131956 10.1038/nprot.2008.211

[36] Goldfarb SB, Kashlan OB, Watkins JN, Suaud L, Yan W, et al (2006) Differential effects of Hsc70 and Hsp70 on the intracellular trafficking and functional expression of epithelial sodium channels. Proc Natl Acad Sci U S A 103: 5817–5822. 16585520 10.1073/pnas.0507903103 PMC1458656

[37] Mariman EC, Schepens JT, Wieringa B (1989) Complete nucleotide sequence of the human creatine kinase B gene. Nucleic Acids Res 17: 6385. 2771648 10.1093/nar/17.15.6385 PMC318286

[38] Feder ME, Hofmann GE (1999) Heat-shock proteins, molecular chaperones, and the stress response: evolutionary and ecological physiology. Annu Rev Physiol 61: 243–282. 10099689 10.1146/annurev.physiol.61.1.243

[39] Kultz D (2005) Molecular and evolutionary basis of the cellular stress response. Annu Rev Physiol 67: 225–257. 15709958 10.1146/annurev.physiol.67.040403.103635

[40] Love S (1999) Oxidative stress in brain ischemia. Brain Pathol 9: 119–131. 9989455 10.1111/j.1750-3639.1999.tb00214.x PMC8098220

[41] Teng J, Takei Y, Harada A, Nakata T, Chen J, et al (2001) Synergistic effects of MAP2 and MAP1B knockout in neuronal migration, dendritic outgrowth, and microtubule organization. J Cell Biol 155: 65–76. 11581286 10.1083/jcb.200106025 PMC2150794

[42] Pan YH, Zhang Y, Cui J, Liu Y, McAllan BM, et al (2013) Adaptation of phenylalanine and tyrosine catabolic pathway to hibernation in bats. PloS one 8: e62039. 23620802 10.1371/journal.pone.0062039 PMC3631164

[43] Srivastava RK, Krishna A (2010) Melatonin modulates glucose homeostasis during winter dormancy in a vespertilionid bat, Scotophilus heathi. Comp Biochem Physiol A Mol Integr Physiol 155: 392–400. 20026417 10.1016/j.cbpa.2009.12.006

[44] Epperson LE, Rose JC, Russell RL, Nikrad MP, Carey HV, et al (2010) Seasonal protein changes support rapid energy production in hibernator brainstem. Journal of comparative physiology B, Biochemical, systemic, and environmental physiology 180: 599–617. 10.1007/s00360-009-0422-9 PMC3116658 19967378

[45] Frerichs KU, Smith CB, Brenner M, DeGracia DJ, Krause GS, et al (1998) Suppression of protein synthesis in brain during hibernation involves inhibition of protein initiation and elongation. Proc Natl Acad Sci U S A 95: 14511–14516. 9826731 10.1073/pnas.95.24.14511 PMC24404

[46] Brown IR (2007) Heat shock proteins and protection of the nervous system. Ann N Y Acad Sci 1113: 147–158. 17656567 10.1196/annals.1391.032

[47] Massa SM, Longo FM, Zuo J, Wang S, Chen J, et al (1995) Cloning of rat grp75, an hsp70-family member, and its expression in normal and ischemic brain. J Neurosci Res 40: 807–819. 7629893 10.1002/jnr.490400612

[48] Xu L, Voloboueva LA, Ouyang Y, Emery JF, Giffard RG (2009) Overexpression of mitochondrial Hsp70/Hsp75 in rat brain protects mitochondria, reduces oxidative stress, and protects from focal ischemia. J Cereb Blood Flow Metab 29: 365–374. 18985056 10.1038/jcbfm.2008.125 PMC3676940

[49] Giffard RG, Xu L, Zhao H, Carrico W, Ouyang Y, et al (2004) Chaperones, protein aggregation, and brain protection from hypoxic/ischemic injury. J Exp Biol 207: 3213–3220. 15299042 10.1242/jeb.01034

[50] Storey KB (1997) Metabolic regulation in mammalian hibernation: enzyme and protein adaptations. Comp Biochem Physiol A Physiol 118: 1115–1124. 9505421 10.1016/s0300-9629(97)00238-7

[51] Eddy SF, McNally JD, Storey KB (2005) Up-regulation of a thioredoxin peroxidase-like protein, proliferation-associated gene, in hibernating bats. Arch Biochem Biophys 435: 103–111. 15680912 10.1016/j.abb.2004.11.020

[52] Lee K, Park JY, Yoo W, Gwag T, Lee JW, et al (2008) Overcoming muscle atrophy in a hibernating mammal despite prolonged disuse in dormancy: proteomic and molecular assessment. J Cell Biochem 104: 642–656. 18181155 10.1002/jcb.21653

[53] Carey HV, Frank CL, Seifert JP (2000) Hibernation induces oxidative stress and activation of NK-kappaB in ground squirrel intestine. J Comp Physiol B 170: 551–559. 11128446 10.1007/s003600000135

[54] Osborne PG, Hashimoto M (2006) Brain antioxidant levels in hamsters during hibernation, arousal and cenothermia. Behav Brain Res 168: 208–214. 16343656 10.1016/j.bbr.2005.11.007

[55] Zhang Y, Pan Y-H, Yin Q, Yang T, Dong D, et al (2014) Critical roles of mitochondria in brain activities of torpid *Myotis ricketti* bats revealed by a proteomic approach. Journal of proteomics 105: 266–284. 24434588 10.1016/j.jprot.2014.01.006

[56] Treiber C, Pipkorn R, Weise C, Holland G, Multhaup G (2007) Copper is required for prion protein-associated superoxide dismutase-I activity in Pichia pastoris. Febs Journal 274: 1304–1311. 17263729 10.1111/j.1742-4658.2007.05678.x

[57] Wong BS, Liu T, Li R, Pan T, Petersen RB, et al (2001) Increased levels of oxidative stress markers detected in the brains of mice devoid of prion protein. J Neurochem 76: 565–572. 11208919 10.1046/j.1471-4159.2001.00028.x

[58] Weissmann C (2004) The state of the prion. Nat Rev Microbiol 2: 861–871. 15494743 10.1038/nrmicro1025

[59] Zomosa-Signoret V, Arnaud JD, Fontes P, Alvarez-Martinez MT, Liautard JP (2008) Physiological role of the cellular prion protein. Vet Res 39: 9. 18073096 10.1051/vetres:2007048

[60] Oliver PL, Finelli MJ, Edwards B, Bitoun E, Butts DL, et al (2011) Oxr1 Is Essential for Protection against Oxidative Stress-Induced Neurodegeneration. PLoS Genet 7. 10.1371/journal.pgen.1002338 PMC3197693 22028674

[61] von der Ohe CG, Darian-Smith C, Garner CC, Heller HC (2006) Ubiquitous and temperature-dependent neural plasticity in hibernators. J Neurosci 26: 10590–10598. 17035545 10.1523/JNEUROSCI.2874-06.2006 PMC6674705

[62] Andrews MT (2007) Advances in molecular biology of hibernation in mammals. Bioessays 29: 431–440. 17450592 10.1002/bies.20560

[63] Millesi E, Prossinger H, Dittami JP, Fieder M (2001) Hibernation effects on memory in European ground squirrels (Spermophilus citellus). J Biol Rhythms 16: 264–271. 11407786 10.1177/074873040101600309

[64] Ruczynski I, Siemers BM (2011) Hibernation does not affect memory retention in bats. Biology letters 7: 153–155. 20702450 10.1098/rsbl.2010.0585 PMC3030893

[65] Harada A, Teng JL, Takei Y, Oguchi K, Hirokawa N (2002) MAP2 is required for dendrite elongation, PKA anchoring in dendrites, and proper PKA signal transduction. Journal of Cell Biology 158: 541–549. 12163474 10.1083/jcb.200110134 PMC2173814

[66] Arendt T, Stieler J, Strijkstra AM, Hut RA, Rudiger J, et al (2003) Reversible paired helical filament-like phosphorylation of tau is an adaptive process associated with neuronal plasticity in hibernating animals. J Neurosci 23: 6972–6981. 12904458 10.1523/JNEUROSCI.23-18-06972.2003 PMC6740664

